# Levels of Cortisol in CSF Are Associated With SNAP-25 and Tau Pathology but Not Amyloid-β

**DOI:** 10.3389/fnagi.2018.00383

**Published:** 2018-11-20

**Authors:** Qing Wang, Wenjun Zhou, Jie Zhang, The Alzheimer’s Disease Neuroimaging Initiative

**Affiliations:** ^1^Wenzhou Seventh People’s Hospital, Wenzhou, China; ^2^Department of Pathology, Hangzhou Normal University, Hangzhou, China; ^3^Independent Researcher, Hangzhou, China

**Keywords:** cortisol, SNAP-25, Alzheimer’s disease, tau pathology, synapse degeneration, mild cognitive impairment

## Abstract

**Objective:** Preclinical studies have found both hyperactivity of hypothalamic- pituitary- adrenal (HPA) axis and synaptic degeneration are involved in the pathogenesis of Alzheimer’s disease (AD). However, the data on the relationship of activity of HPA axis and synaptic degeneration in humans are limited.

**Methods:** We compared CSF cortisol levels in 310 subjects, including 92 cognitively normal older people, 149 patients with mild cognitive impairment (MCI), and 69 patients with mild AD. Several linear and logistic regression models were conducted to investigate associations between CSF cortisol and synaptosomal-associated protein 25 (SNAP-25, reflecting synaptic degeneration) and other AD-related biomarkers.

**Results:** We found that levels of cortisol in CSF were associated with SNAP-25 levels and tau pathologies but not amyloid-β protein. However, there were no significant differences in CSF cortisol levels among the three diagnostic groups.

**Conclusion:** The HPA axis may play a crucial role in synaptic degeneration in AD pathogenesis.

## Introduction

Alzheimer’s disease (AD) is a common neurodegenerative disorder characterized by a progressive decline of memory and cognitive function (Scheltens et al., 2016). Although it is well known that amyloid plagues and neurofibrillary tangles are two key neuropathological features in AD, the mechanisms underlying the pathogenesis of AD are not fully understood. A growing body of evidence support the idea that the hyperactivity of hypothalamic- pituitary- adrenal (HPA) axis with excess cortisol may be involved in cognitive impairment and dementia (Csernansky et al., 2006; Lee et al., 2007; Gil-Bea et al., 2010). In addition, it has been reported that the degree of hyperactivity of the HPA-axis was associated with the severity of hippocampal atrophy and cognitive impairment in patients with dementia (de Leon et al., 1988;Gurevich et al., 1990). Animal studies reported that chronic stress and glucocorticoids can accelerate memory decline and increase amyloid-β and tau pathologies in mouse models of AD (Green et al., 2006; Jeong et al., 2006). Further, the authors also found that elevated glucocorticoid levels increase Aβ production by increasing levels of amyloid precursor protein (APP) and β-APP cleaving enzyme (BACE) (Jeong et al., 2006). Taken together, these findings highlight an important role of cortisol in the pathogenesis of AD.

Synaptic function has been regarded as a critical mechanism underlying cognitive impairment in the pathogenesis of AD (Selkoe, 2002). A previous study suggested that presynaptic proteins (e.g., synaptobrevin, synaptotagmin, and Rab3a) were significantly decreased in AD brains (Sze et al., 2000). Synaptosomal-associated protein 25 (SNAP-25) is a presynaptic protein which plays an important role in neuronal survival and cognition (Toonen et al., 2017). One previous study showed that SNAP-25 was elevated in the CSF of patients with mild cognitive impairment (MCI) and AD, indicating that SNAP-25 may be a useful biomarker reflecting synapse degeneration (Brinkmalm et al., 2014; Zhang et al., 2018). Importantly, it has been demonstrated that corticosterone can cause a substantial loss of synapses in rodents, indicating a potential role of corticosterone in the maintenance of synapse function (Tata et al., 2006). To our knowledge, no human studies on the association between CSF cortisol levels and synapse degeneration have been reported.

Here, the levels of cortisol in CSF among normal controls, patients with MCI and AD had been compared. Second, we examined the relationship of CSF cortisol levels and synapse degeneration. Finally, we investigated the associations between CSF cortisol levels and typical AD-related biomarkers, including hippocampal volumes, CSF Aβ42, t-tau, and p-tau levels.

## Materials and Methods

### Alzheimer’s Disease Neuroimaging Initiative

Data used in the preparation of this study were extracted from the Alzheimer’s Disease Neuroimaging Initiative (ADNI) database. The primary goal of ADNI has been to investigate whether neuropsychological testing, neuroimaging data, blood, and CSF biomarkers could be integrated to assess the progression of MCI and mild AD. This study was approved by local ethical committees. All subjects or authorized representatives provides written informed consent. Further information can be found at the website: http://www.adni-info.org.

### Participants

Demographics were obtained from the ADNI database. In this analysis, there were 92 normal controls, 149 patients with MCI, and 69 patients with mild AD.

### APOE Genotyping, Clinical and Memory Assessments

APOE genotypes of the study participants were extracted from the ADNI database^1^. Participants underwent comprehensive cognitive assessments, while we only selected Mini-Mental State Exam (MMSE) and Rey Auditory Verbal Learning Test (RAVLT immediate recall and delayed recall) in the current analysis.

### Cerebrospinal Fluid Data

#### Quantification of Cortisol in CSF

The levels of CSF cortisol were determined using Luminex xMAP immunoassay technology, which measures a range of inflammatory, lipid, metabolic, and other indices (Olsson et al., 2013). Further information can be found at the website: http://www.adni-info.org. The CSF cortisol data were extracted from the ADNI database. Value are given in ng/ml.

#### Quantification of SNAP- 25 in CSF

Levels of SNAP-25 in CSF were determined at the departments of pathology and neurology at Washington University, St. Louis, United States. A sandwich ELISA that was developed using the Erenna^®^ immunoassay system to determine the levels of SNAP-25 in CSF. All samples were run in triplicates. Values are given as pg/ml. The detailed protocol can be found at http://www.adni-info.org.

#### Quantification of Aβ42, t-tau, and p-tau in CSF

Levels of Aβ42, t-tau, and p-tau in CSF were determined by the ADNI Core Biomarkers Team using xMAP Luminex platform and Innogenetics/Fujirebio AlzBio3 immunoassay kits. The detailed procedure for this determination is described elsewhere (Shaw et al., 2009). Using a previously proposed CSF Aβ42 cutoff < 192 pg/ml (Shaw et al., 2009), participants were classified as abnormal or normal. The ROC analysis found that this cutoff value can detect the autopsy-confirmed AD cases with a high sensitivity (96.4%) (Shaw et al., 2009). Tau phosphorylated at the threonine 18 was measured.

### Hippocampal Volumes

The hippocampal volumes data were extracted from the ADNI database. The detailed neuroimaging methods used by ADNI have been described elsewhere (Zhang et al., 2011). Briefly, structural MRI brain scans were obtained using 1.5T MRI scanners with a standardized protocol, which is described in detail at adni.loni.usc.edu. Hippocampal volume measurements were performed using FreeSurfer software^2^.

### Statistical Analysis

First, the *F*-test and *x*^2^ test were performed to investigate the mean differences of continuous variables and the distributions of categorical variables, respectively. Second, the Pearson correlation tests were used to investigate the correlations of CSF cortisol levels and other AD-related biomarkers in all participants. Finally, linear and logistic regression models were used to examine the association between cortisol and other biomarkers after adjusting several potential confounding variables. More specifically, logistic regression models were used to examine the association between amyloid status (<192 pg/ml vs. >192 pg/ml; dependent variable) and CSF cortisol levels (independent variable). Model 1 was unadjusted. Model 2 was adjusted for age, education, age, APOE genotype, MMSE scores and diagnosis. All statistical analyses were performed using R software. A two tailed *P* < 0.05 was considered to be statistically significant.

## Results

### Sample Characteristics

Table 1 describes the demographic and clinical information: 92 normal controls, 149 patients with MCI, and 69 patients with AD. No significant differences in age and education were found across the three diagnostic groups. Expectedly, a significant difference in MMSE scores across the three diagnostic groups was detected (*p* < 0.001). In addition, there were significant differences in RAVLT immediate and delayed recall scores across the three diagnostic groups (*p* < 0.001). Consistent with the previous data, more than 50% of patients with MCI and AD carried at least one APOE 4 allele (Farrer et al., 1997). There were significant differences in CSF SNAP-25, Aβ42, t-tau, p-tau, and hippocampal volumes across the three diagnostic groups (*P* < 0.005). However, no significant differences in CSF cortisol levels were found between the three groups (*P* > 0.05).

**Table 1:** Demographic and clinical variables.

Clinical variables	NC (*n* = 92)	MCI (*n* = 149)	AD (*n* = 69)	*P*-value
Age, years	75.7 ± 5.4	74.8 ± 7.2	74.9 ± 7.6	0.622
Female/ Male, *n*	46/46	47/102^$^	30/39	0.013
APOE 4 carriers/ noncarriers, *n*	22/70	80/69^$^	49/20^&,∗^	<0.001
Education, years	15.6 ± 2.9	16 ± 2.9	15.2 ± 3	0.18
MMSE	29.1 ± 1	26.9 ± 1.8^$^	23.5 ± 1.8^&,*^	<0.001
RAVLT-immediate recall	43 ± 8.6	29.6 ± 8.6^$^	23.1 ± 7.4^&*^	<0.001
RAVLT-delayed recall	6 ± 2.2	3.3 ± 2.3^$^	2 ± 1.6^&,*^	<0.001
Hippocampal volume, cm^3^	7.2 ± 0.8	6.3 ± 1^$^	5.8 ± 1.3^&,*^	<0.001
CSF cortisol, ng/ml	15.6 ± 6.2	16.8 ± 5.8	15.7 ± 6.2	0.22
CSF SNAP-25, pg/ml^a^	4.5 ± 1.4	5.6 ± 2.3^$^	6.1 ± 1.7^*^	0.002
CSF Aβ42, pg/ml	208 ± 53.4	160 ± 49.2^$^	141.4 ± 35^&,*^	<0.001
CSF t-tau, pg/ ml	68.8 ± 26.4	105.5 ± 52.8^$^	122.2 ± 59^&,*^	<0.001
CSF p-tau, pg/ml	24.8 ± 13.4	36.3 ± 15.5^$^	41.1 ± 20.6^*^	<0.001

*NC, normal controls; MCI, mild cognitive impairment; AD, Alzheimer’s disease; MMSE, mini-mental state examination; SNAP-25, synaptosomal-associated protein 25; t-tau, total tau protein; p-tau, phospho-tau protein; Aβ42, β-amyloid 42. Values are expressed as mean ± standard deviation. ^a^Included in this analysis are 143 subjects, including 55 NC, 73 MCI, and 15 AD subjects. Comparison between NC group and MCI group is marked behind “MCI group,” ^$^p < 0.05. Comparison between MCI group and AD group is marked behind “AD group,” ^&^p < 0.05. Comparison between NC group and AD group is marked behind “AD group,” ^∗^p < 0.05.*

### CSF Cortisol Levels in the Three Groups

**FIGURE 1:**
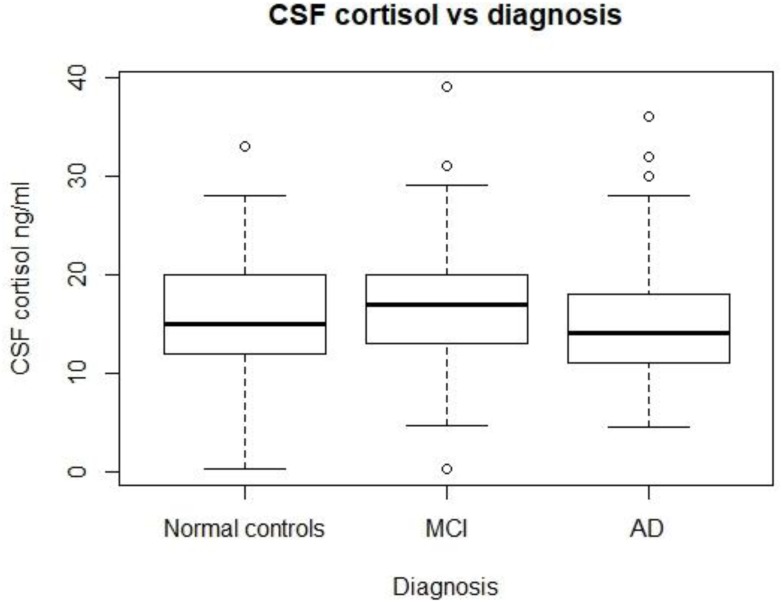
CSF cortisol levels in three diagnostic groups. There are no significant differences in CSF cortisol between the three diagnostic groups (*p* > 0.05). MCI, mild cognitive impairment; AD, Alzheimer’s disease.

As Figure 1 shows, no significant differences in CSF cortisol levels were found across three groups (*p* > 0.05). In this analysis, we included 310 subjects, including 92 normal controls, 149 patients with MCI, and 69 patients with mild AD.

### Correlations Between CSF Cortisol Levels and SNAP- 25, Aβ42, t-tau and p-tau Levels

**FIGURE 2:**
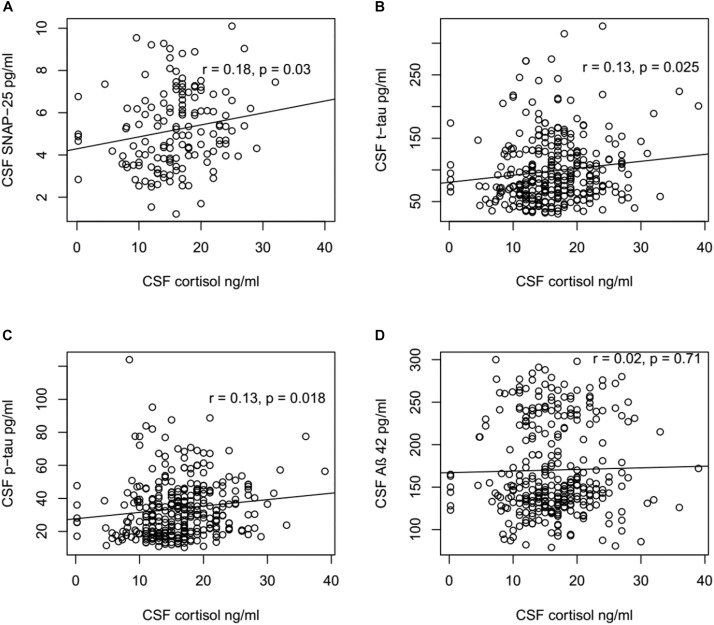
Correlation between CSF cortisol levels and SNAP-25, Aβ42, t-tau, and p-tau levels. **(A)** CSF cortisol levels were positively associated with SNAP-25 in all participants (*r* = 0.18, *p* = 0.03). **(B)** CSF cortisol levels were positively associated with t-tau in all participants (*r* = 0.13, *p* = 0.025). **(C)** CSF cortisol levels were positively associated with p-tau in all participants (*r* = 0.13, *p* = 0.018). **(D)** No significant relationship existed between CSF cortisol levels and Aβ42 in all participants (*r* = 0.02, *p* = 0.71). **(A)** Included in this analysis are 143 subjects, including 55 NC, 73 MCI, and 15 AD subjects. **(B–D)** Included in this analysis are 310 subjects, including 92 NC, 149 MCI, and 69 AD subjects.

To explore the mechanism underlying the role of cortisol in the pathogenesis of AD, the correlations between CSF cortisol and other AD-related biomarkers in all participants were analyzed using Pearson correlation tests (Figure 2). A positive correlation between CSF cortisol and SNAP-42 levels was observed (*r* = 0.22, *p* = 0.009). Additionally, the correlations between CSF cortisol between tau protein were analyzed. Levels of cortisol in CSF were associated with CSF t-tau (*r* = 0.13, *p* = 0.025) and p-tau (*r* = 0.13, *p* = 0.018) levels. However, no correlations between CSF cortisol and Aβ42 levels were found (*p* > 0.05, Figure 2).

### Linear and Logistic Regression Results

We examined the associations between CSF cortisol and memory outcomes, synapse degeneration (SNAP-25), and AD-related biomarkers (hippocampal volumes, Aβ42, tau protein). Higher levels of cortisol in CSF were significantly associated with greater SNAP-25, t-tau, p-tau levels (*p* < 0.05, Table 2). After controlling for covariates in model 2, the associations between cortisol and SNAP -25, t-tau, p-tau levels remained (Table 2). However, CSF cortisol levels were not significantly associated with performance on tests of memory and hippocampal volumes (Table 2). In addition, we evaluated the relationship between cortisol and amyloid status (<192 pg/ml vs. >192 pg/ml) using logistic regression model, but the results did not reach significance.

**Table 2:** Association between CSF cortisol and cognition and other biomarkers.

CSF cortisol levels^a^	Model 1		*P*-value	Model 2		
	*n*	B (95% CI)		*n*	B (95% CI)	*P*-value

Linear regression models			
RAVLT-immediate recall	309	-0.15(-0.36 – 0.06)	0.15	309	-0.05(-0.21 – 0.1)	0.49
RAVLT-delayed recall	297	-0.01(-0.06 – 0.04)	0.66	297	0.01(-0.04 – 0.05)	0.75
Hippocampal volumes^b^, mm^3^	251	-21(-47 – 5)	0.11	251	-7.5(-27 – 12)	0.45
CSF SNAP-25	143	0.07(0.02 – 0.13)	0.009	143	0.08(0.03 – 0.13)	0.003
CSF t-tau	310	1.1(0.14 – 2.1)	0.025	310	1.3 (0.4 – 2.2)	0.006
CSF p-tau	310	0.39(0.07 – 0.7)	0.018	310	0.45(0.15 – 0.76)	0.004

**CSF cortisol levels**	**Model 1**		***P*-value**	**Model 2**		***P*-value**
	***n***	**OR (95% CI)**		***n***	**OR (95% CI)**	

**Logistic regression model**			

Abnormal CSF Aβ levels^c^	310	0.99(0.96 – 1.04)	0.78	310	0.98(0.93 – 1.04)	0.55

*OR, odds ratio; CI, confidence interval. Model 1 is unadjusted. Model 2 was adjusted for age, gender, education, APOE 𝜀4, MMSE scores, and diagnosis. ^a^CSF cortisol was treated as independent variable in linear regression models. ^b^Model 2 was adjusted for age, gender, education, diagnosis, APOE 𝜀4, MMSE scores, and intracranial volume (ICV). ^c^Abnormal CSF Aβ levels (<192 pg/ml) was treated as dependent variable in logistic regression models.*

## Discussion

To the best of our knowledge, the current study was the first to investigate the relationship of CSF cortisol levels and synapse degeneration among cognitively normal older people, patients with MCI and AD. In addition, we also found that levels of cortisol in CSF were associated with tau pathologies but not amyloid-β protein. However, no significant differences in CSF cortisol levels were found among the three diagnostic groups.

Epidemiological studies suggested that chronic environmental stress plays an important role in the pathogenesis of AD (Pardon, 2011; Zverova et al., 2013). Additionally, preclinical studies have found that glucocorticoids and corticotrophin-releasing hormone (CRH) were involved in AD pathogenesis by manipulating the levels of Aβ and tau pathologies (Kulstad et al., 2005; Green et al., 2006; Jeong et al., 2006; Filipcik et al., 2012; Rothman et al., 2012). Conversely, Baglietto-Vargas et al. (2013) reported that mifepristone, a glucocorticoid receptor antagonist, can reduce levels of Aβ and tau and ameliorate cognitive deficits in mice models of AD. Taken together, these findings highlight an important role of chronic stress and stress-related hormones in affecting AD pathogenesis. In the present study, we found that levels of cortisol in CSF were positively correlated with tau pathology and synapse degeneration in normal controls, subjects with MCI and AD. Our findings were consistent with the results from previous studies (Green et al., 2006; Sotiropoulos et al., 2011). For instance, it has been reported that chronic stress can trigger tau hyperphosphorylation, a core mechanism in AD, and lead to cognitive impairment (Green et al., 2006; Sotiropoulos et al., 2011). Importantly, tau hyperphosphorylation plays a key role in synaptic function and neuronal survival related with AD due to the fact that phosphorylated tau destabilizes microtubules and results in tau accumulation and cytoskeletal damage (Fitzpatrick et al., 2017). In addition, tau mislocalization to synapses may be associated with synaptic dysfunction in AD (Hoover et al., 2010; Tai et al., 2012). These findings could possibly explain how higher levels of cortisol in brain lead to synapse dysfunction, and ultimately cognitive deficits. Although numerous evidence suggested an association of cortisol with synaptic function in preclinical studies, the data in human are limited. Our data found that CSF cortisol was associated with synaptic degeneration in normal controls, patients with MCI and AD. These findings strengthen the idea that CSF cortisol may be associated with synapse degeneration. However, further studies are needed to explore the mechanisms that underlie the associations of cortisol and synaptic degeneration.

In the present study, no significant differences in CSF cortisol levels were observed among three diagnostic groups. It has been reported that there were no differences in cortisol levels in plasma (Csernansky et al., 2006), salivary (Wolf et al., 2002; Souza-Talarico et al., 2010) or CSF (Popp et al., 2009) between cognitively normal subjects and patients with MCI. In contrast, others found greater levels of cortisol in salivary in patients with MCI (Arsenault-Lapierre et al., 2010). However, in patients with AD, previous studies reported elevated levels of cortisol in plasma, salivary, urinary and (Greenwald et al., 1986; Peskind et al., 2001; Rasmuson et al., 2001; Hoogendijk et al., 2006; Curto et al., 2017). These inconsistencies in the published literature on cortisol levels in MCI or AD may result from several differences between studies, including: (1) the sample, which can be salivary, plasma, urinary and CSF; (2) sample size, which can be vary from a handful of participants to many 100’s of participants; and (3) methodological differences in the assay procedure which may have effects on sensitivity.

In present study, we did not find significant correlations between CSF cortisol and Aβ-42 levels, which is consistent with a previous study (Laske et al., 2009). However, in the same study, Laske et al. (2009) found that cortisol levels in serum, but not CSF, were associated with CSF Aβ-42 levels, suggesting serum and CSF cortisol levels appear to be independent in AD patients. Recently, one study investigating the relationship of serum cortisol and amyloid metabolism in patients with major depressive disorder (MDD) found that serum cortisol levels were not associated with serum Aβ levels at baseline. However, cortisol levels in serum at baseline were found to be correlated with serum Aβ levels 1 year later (Ishijima et al., 2018). The precise association of cortisol and amyloid metabolism needs further investigation.

Several limitations should be noted. First, cross-sectional design limits our ability to explore the temporal relationship of cortisol and synapse degeneration and AD-related biomarker. Second, the findings of this study may not be generalizable to other populations. Finally, this study included only subjects with mild AD. Further study should also include moderate and severe dementia patients to clarify the relationship of severity of cognitive impairment and CSF cortisol levels.

## Conclusion

In conclusion, we found that CSF cortisol levels were associated with tau pathologies and synapse degeneration but not amyloid-beta, highlighting a potential role of cortisol in synapse degeneration in AD patients.

## Author Contributions

JZ conceived and designed the studies. QW, WZ, and JZ performed the research, analyzed the data, and wrote the manuscript.

## Conflict of Interest Statement

The authors declare that the research was conducted in the absence of any commercial or financial relationships that could be construed as a potential conflict of interest.

## Footnotes

1 adni.loni.usc.edu

2 http://surfer.nmr.mgh.harvard.edu
